# P-923. Characterization of the use of the FilmArray Molecular panel for the diagnosis of central nervous system infection

**DOI:** 10.1093/ofid/ofae631.1114

**Published:** 2025-01-29

**Authors:** Silvana zapata, Daniel Montoya Roldan, Pablo Villa franco, Sandra patricia isaza, Santiago Atehortua, Carolina Zapata, Carolina Lopez estrada, Iván Trompa

**Affiliations:** Universidad de Antioquia, Medellin, Antioquia, Colombia; Hospital Alma Mater, Medellín, Antioquia, Colombia; Hospital Pablo Tobón Uribe, medellin, Antioquia, Colombia; Universidad de Antioquia, Medellin, Antioquia, Colombia; Hospital Pablo Tobón Uribe, medellin, Antioquia, Colombia; Hospital Alma Mater, Medellín, Antioquia, Colombia; Universidad Pontificia Bolivariana, Medellin, Antioquia, Colombia; Hospital Alma Mater, Universidad de Antioquia, Medellin, Antioquia, Colombia

## Abstract

**Background:**

The Biofire FilmArray® Meningitis/Encephalitis Panel (FAME) has proven to be a valuable tool for etiological diagnosis. Despite this, we know little about its clinical impact.This investigation aims to characterize the clinical, epidemiological, therapeutic variables and outcomes of adults with suspected meningitis and/or encephalitis (ME) who underwent FAME panel, in two 3^rd^ level hospitals in Medellín-Colombia.

Central nervous system infection diagnosis

**Figure:**
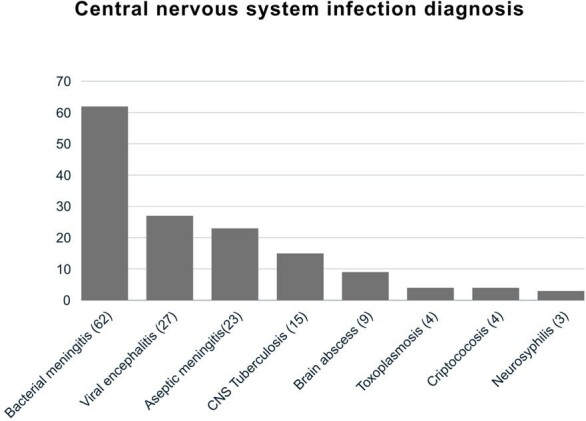

Definitive diagnosis based on clinical and microbiologic criteria

**Methods:**

Retrospective cohort study in 257 patients over 16 years of age, with suspected central nervous system infection with less than 4 weeks of symptoms who underwent FAME panel. Comparisons were made between those with positive and negative FAME panel results, and a medical record evaluation committee determined whether central nervous system infection was present.

Identification of pathogens by Film Array meningitis encephalitis

**Figure:**
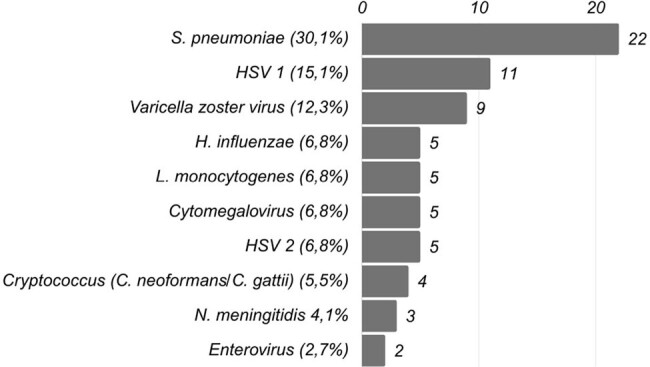

Pathogens identified

**Results:**

57.19% (147) of the patients presented central nervous system infection, with 70 patients (26.07%) with a positive FAME panel result; the most frequent involvement being bacterial meningitis 37 (52.9%), and among those with negative FAME panel it was aseptic meningitis 23 (15.64%) followed by meningeal tuberculosis 15 (10.2%). Altered consciousness (74.3%), fever (63.4%) and headache (63.4%) predominated in the FAME-positive group, as did higher cerebrospinal fluid (CSF) leukocyte count, glucose consumption and hyperproteinorrachia (p =0.0001). A positive FAME panel result was associated with greater modification of empirical to definitive antibiotic therapy and discontinuation of unnecessary antibiotics, while negative results were associated with shorter hospital stays, fewer neurological sequelae, and lower antibiotic consumption.

Of the total number of patients with positive FAME, 9 had a CSF leukocytes of less than 5 cells/mm3. Additionally, of the total, eight (8) had pharmacological or disease-related immunosuppression, and only one case had a positive FAME for Varicella zoster without medical or pharmacological immunosuppression with normal CSF.

**Conclusion:**

FAME panel in the appropriate context can provide valuable information for decision-making in patients with suspected central nervous system infection; a rational use and restrictive criteria in prescription could improve the benefits without incurring in excessive costs.

**Disclosures:**

**All Authors**: No reported disclosures

